# Supplementary material for: Serum anti-erythropoietin antibodies among pregnant women with Plasmodium falciparum malaria and anaemia: A case-control study in northern Ghana
Source: PLoS One. 2023 Mar 29;18(3):e0283427. doi: 10.1371/journal.pone.0283427 (PMC10058094; doi:10.1371/journal.pone.0283427)
Supplement: Attachment — Submitted filename: RESPONSE TO REVIEWERS.docx [file pone.0283427.s001.docx]

Dear Editor,

Thank you for your email. I am pleased to resubmit manuscript titled “**Serum anti-erythropoietin antibodies among pregnant women with *Plasmodium falciparum* malaria and anaemia: A case-control study in northern Ghana** “for your consideration.

Authors do appreciate your endless efforts to ensure improvement in the manuscript.

The concerns raised by the editorial team have been addressed and highlighted below and marked in the respective sections in the manuscript. I look forward to your favourable response.

Thank you.

**The manuscript was edited for language usage, spelling and grammar by Grammarly Inc. USA.**

**General Comments**

1. Initial incorrect spelling of the middle name of the last author

Response: Authors middle name is correctly spelt: **Ejike.** This makes the full name **Felix Ejike Chukwurah**

2. Kindly add the city and country of the manufacturer of the statistical software you used for analysis.

Response: This has been considered and inputs made

3. Also, give the full meaning of the abbreviations at first use.

Response: This has been considered throughout the document

**Reviewer #1:**

1. authors should state categorically how Plasmodium falciparum density and age relates to anti-EPO do anti-EPO enhanced or inhibit anti-EPO

Response: *P. falciparum* density and younger age may stimulate the production of anti-EPO in pregnant women with malaria

2. Line 59 sentence .......... in both foetal and or maternal lives should be revised/rephrased to enhanced clarity

Response: This has been revised accordingly

3. Line 100 define EPO first time usage

Response: EPO is first defined in line 37 of the manuscript.

4. Line 74-76 ........but suggested via multiple mechanisms including suppression .....revise to enhance clarity of understanding.

Response: This has been revised to ensure clarity

5. Sample size calculation as demonstrated in the manuscript is too detailed, authors should just state the name of the formulae, the prevalence used, referenced the articled from which it was sourced from and finally the sample size itself.

Response: The detailed presentation of the sample size determination will allow for reproducibility.

6. how did authors ensured that exclusion criteria were met ie P. falciparum-infected pregnant women with comorbidities (such as infections and other haematological disorders) were excluded.

Response: Clinical characteristics of the pregnant women (participants) were obtained from the ante-natal care (ANC) register, which contained results on HIV, HBsAg, HCV, Syphilis, Sickling test, Hb phenotype, G6PD test, routine examinations of stool and urine, etc. Pregnant women with comorbidities were excluded from the study, as shown in lines 149-150.

7. line 100 subheading assessment of CBC should be changed to Measurement of CBC

Response: The subheading has been revised to read ‘Measurement of complete blood count’, and seen in line 165

8. Authors failed to demonstrate how they speciated P. falciparum from other Spp. of anopheles

Response: This has been addressed and inserted in lines 198 to 205

9. Authors should move the limitation of the study from conclusion section to the discussion section

Response: The limitation of the study has been moved from the conclusion to the discussion section as recommended.

**Reviewer #2:**

The animal studies in the narration may not directly substitute for pregnant humans. The findings with animal studies that were not pregnant is far too wide for comparative analysis. The study is on human pregnant conditions, P. Falciparum and presence of antiEOP antibodies. It may not be absolutely necessary in the discussion page of this study.

Response: This has been considered and revision made
