# Supplementary material for: Serum anti-erythropoietin antibodies among pregnant women with Plasmodium falciparum malaria and anaemia: A case-control study in northern Ghana
Source: PLoS One. 2023 Mar 29;18(3):e0283427. doi: 10.1371/journal.pone.0283427 (PMC10058094; doi:10.1371/journal.pone.0283427)
Supplement: Attachment — Submitted filename: RESPONSE TO REVIEWERS.docx [file pone.0283427.s002.docx]

Dear Editor,

Thank you for your email. I am pleased to resubmit manuscript titled “**Serum anti-erythropoietin antibodies among pregnant women with *Plasmodium falciparum* malaria and anaemia: A case-control study in northern Ghana** “for your consideration.

Authors do appreciate your endless efforts to ensure improvement in the manuscript.

The concerns raised by the editorial team have been addressed and highlighted below and marked in the respective sections in the manuscript. I look forward to your favourable response.

Thank you.

**The manuscript was edited for language usage, spelling and grammar by Grammarly Inc. USA.**

**General Comments**

1. Kindly revise line 94 in the introduction. Suggestion: Delete the sentence completely as you did in the discussion section (i.e comparison with animal-mice), since your work is on humans it won't be appropriate referring to an animal study.

Response: The entire sentence has been deleted as suggested

2. In Table 1, remove the word 'years' after the different ages groups since you have written it in bracket in the preceding row. In Table 5 format as suggested above for Table 1.Initial incorrect spelling of the middle name of the last author

Response: The word ‘years’ has been deleted from both Tables 1 and 5 accordingly**.**

**Reviewer #2:**

The animal studies in the narration may not directly substitute for pregnant humans. The findings with animal studies that were not pregnant is far too wide for comparative analysis. The study is on human pregnant conditions, P. Falciparum and presence of anti-EOP antibodies. It may not be absolutely necessary in the discussion page of this study.

Response: This has been considered and revision made
